# Using Twitter for breast cancer prevention: an analysis of breast cancer awareness month

**DOI:** 10.1186/1471-2407-13-508

**Published:** 2013-10-29

**Authors:** Rosemary Thackeray, Scott H Burton, Christophe Giraud-Carrier, Stephen Rollins, Catherine R Draper

**Affiliations:** 1 Department of Health Science, Brigham Young University, 221Richards Building, Provo, UT 84602, USA; 2 Department of Computer Science and Electrical Engineering, Brigham Young University-Idaho, 216 Austin building, Rexburg, ID 83460, USA; 3 Department of Computer Science, Brigham Young University, 3361 TMCB, Provo, UT 84602, USA

**Keywords:** Social media, Breast cancer, Campaign, Twitter, Awareness

## Abstract

**Background:**

One in eight women will develop breast cancer in her lifetime. The best-known awareness event is breast cancer awareness month (BCAM). BCAM month outreach efforts have been associated with increased media coverage, screening mammography and online information searching. Traditional mass media coverage has been enhanced by social media. However, there is a dearth of literature about how social media is used during awareness-related events. The purpose of this research was to understand how Twitter is being used during BCAM.

**Methods:**

This was a cross-sectional, descriptive study. We collected breast cancer- related tweets from 26 September - 12 November 2012, using Twitter’s application programming interface. We classified Twitter users into organizations, individuals, and celebrities; each tweet was classified as an original or a retweet, and inclusion of a mention, meaning a reference to another Twitter user with @username. Statistical methods included ANOVA and chi square. For content analysis, we used computational linguistics techniques, specifically the MALLET implementation of the unsupervised topic modeling algorithm Latent Dirichlet Allocation.

**Results:**

There were 1,351,823 tweets by 797,827 unique users. Tweets spiked dramatically the first few days then tapered off. There was an average of 1.69 tweets per user. The majority of users were individuals. Nearly all of the tweets were original. Organizations and celebrities posted more often than individuals. On average celebrities made far more impressions; they were also retweeted more often and their tweets were more likely to include mentions. Individuals were more likely to direct a tweet to a specific person. Organizations and celebrities emphasized fundraisers, early detection, and diagnoses while individuals tweeted about wearing pink.

**Conclusions:**

Tweeting about breast cancer was a singular event. The majority of tweets did not promote any specific preventive behavior. Twitter is being used mostly as a one-way communication tool. To expand the reach of the message and maximize the potential for word-of-mouth marketing using Twitter, organizations need a strategic communications plan to ensure on-going social media conversations. Organizations may consider collaborating with individuals and celebrities in these conversations. Social media communication strategies that emphasize fundraising for breast cancer research seem particularly appropriate.

## Background

Media coverage of breast cancer, including breast cancer awareness month (BCAM) and associated events has been a key component to increasing awareness of breast cancer and rates of screening mammograms. Today, the spread of the breast cancer message is no longer limited to traditional media outlets because individuals have access to several social media platforms for both finding and sharing information. One popular social media application is Twitter. On Twitter, individuals and organizations can post (i.e. tweet) their thoughts, ideas, reactions to events, and so forth in 140 characters or less. Twitter users can follow each other to receive a real-time feed of the users’ respective tweets. Users can also pass others’ messages on to their followers (i.e. retweet), as well as make explicit reference to others by username (i.e. mention), which puts the tweet into an additional subscription feed. Researchers estimate that among adults who go on-line, 18% have Twitter accounts [1]. Both men and women are equally likely to use Twitter, but adults less than 30 years old, Blacks and Hispanics, and people living in urban settings have higher usage rates [1].

Unless the Twitter users mark tweets as private, tweets are public, making Twitter a rich source of information about the thoughts of a broad range of people regarding a variety of topics including breast cancer. Twitter has been used for several health-related purposes, including to disseminate information about diabetes [2], communicate during a disaster [3]and to understand health-related trends and issues such as influenza [4], tobacco [5], problem drinking [6], dental pain [7], antibiotics and prescription drug misuse [8,9], and others [10].

In a study of how organizations use the social networking site Facebook for cancer awareness and community building, researchers found that five key activities were: to inform and educate, provide support, share testimony, advocate, and raise funds. These functions may be the same for cancer organizations using Twitter [11]. However, there is a dearth of literature about how social media, particularly, Twitter, is being used to increase awareness about health issues, including those identified as part of national health observances (NHO) which are days, weeks, or months dedicated to a focus on specific health topics in the United States. In the early 1990s the United States government recognized the month of October as an official national health observance for breast cancer awareness [12].

This paper is presented as a case study of how Twitter was used during BCAM, an official NHO. The purposes of this research were to understand the use of Twitter during BCAM by answering the following questions:

•What is the frequency of tweeting about breast cancer during BCAM?

•Are individuals, organizations, or celebrities more likely to tweet about BCAM?

•What is the reach of messages about BCAM?

•What is the content of tweets during BCAM?

It is estimated that one in eight women will develop breast cancer in her lifetime [13]. In 2013 alone, over 39,000 women will die from breast cancer [14].While mammography is effective at early detection of breast cancer tumors [15], the frequency and age at which women should start screening mammography is controversial. The American Cancer Society recommends that all women over 40 receive an annual clinical breast exam and a screening mammogram [15]. The U.S. Preventive Task Force recommends screening every two years, starting at age 50 [16]. Not everyone agrees that breast cancer mammography screening is necessary or effective at reducing breast cancer mortality and may actually do more harm than good [17]. A review of randomized control trials showed that screening mammography reduced breast cancer mortality [18]. However, screening mammography also lead to a 30% overdiagnosis and overtreatment. This means that women were diagnosed with breast cancer who did not have it and they subsequently received unnecessary treatment for breast cancer.

The annual BCAM has traditionally focused on increasing women’s participation in early detection through mammograms, along with educating women about breast cancer and increasing general awareness of breast cancer [12,19]. Events typically associated with BCAM include walks and sporting events, lectures, display of posters and other communication materials, and “wear pink” days [12]. These outreach efforts have resulted in increased media coverage of breast cancer, especially during October [12].

This media coverage has led to increased rates of breast cancer screening among women. In early 1987, just after the American Cancer Society started their focus on breast cancer awareness, but before it became an official NHO, only 26% of women in the United States had undergone a mammogram in the previous 12 months; by October of the same year the proportion had reached 38% [12]. Rates continued to increase and by 1999, about 70% of women had received a mammogram in the last two years [12]. Breast cancer screening rates have only slightly increased in the last decade, with approximately 72.4%-77.9% of women ages 50 and older having received a mammogram in the last two years [20,21]. Another study verified that BCAM was associated with increased breast cancer screening. Researchers examined rates of breast cancer detection across 97 quarters that included BCAM, from 1975–1997. The results showed there was an increase in the rate of breast cancer detection during the time period that included BCAM [22].

BCAM is also associated with increased online searching of information about breast cancer. A study by Glynn and colleagues [23] showed that during October (i.e. BCAM) there were more on-line searches for the topic of breast cancer than during other times of the year. This same trend did not hold for searching for prostate cancer or lung cancer during their respective awareness months. This indicates that over two decades of BCAM activities have resulted in significant awareness levels of breast cancer as a leading cause of death among women.

## Methods

This was a cross-sectional, descriptive study. We collected breast cancer related tweets from Twitter from five days before the start of BCAM in October 2012 (September 26) to 12 days after (November 12). Twitter provides an application programming interface (API) that enables automatic consumption of tweets as they are posted in real-time. Using the terms listed below, we used the Twitter API to filter the general Tweet-stream, to obtain only those tweets that contained keywords relevant to BCAM. There were 1,744,271 tweets from 1,013,104 unique users.

### Filter terms used to obtain breast cancer tweets from Twitter

Pink ribbon, Wear pink, wearpink, Mammogram, Breast cancer, Breastcancer, breast screening, breast Tumor, breast cure, Susan Komen, Susan Comen, Race for the cure, bcaware, survivorship breast, SusanGKomen, raceforthecure, beatcancer, pinkribbon, self breast exam, self breast examination, codepink, code pink, lump breast, breast chemo, lumpectomy, mastectomy, breast standup, stand up breast, livestrong breast, standuptocancer breast, breastcancerawareness, breastcancersurvivor, breastcancersurvivors, pinkarmy, bcsm.

An initial analysis of the distribution of tweets over time revealed an unexpected spike in the frequency of tweets posted on Wednesday, October 3. Investigating the tweets on this day revealed that a second group of unrelated tweets using keywords such as “wear”, “pink”, and “Wednesday” had been picked up along with the breast cancer tweets. These tweets expressed a motivation for adolescent school girls to wear pink clothing to identify themselves as “Mean Girls” (from the popular 2004 movie of the same name, starring actress Lindsay Lohan) completely independent of BCAM (e.g., “We wear pink on Wednesdays if you don’t you cant sit with us #MeanGirls” [sic.]). Using a second set of filter terms (i.e. mean girls, wear pink wednesday(s), wear pink wed(s)), the initial set of breast cancer related tweets was refined to exclude these unrelated tweets for a total of 1,574,332 tweets from 899,764 users. For all remaining analyses, we restricted the data to October 1- October 31, the actual days for BCAM, resulting in 1,351,823 tweets from 797,827 users. During this time period, there was an average of a half-billion tweets per day [24,25], so BCAM tweets represented about .27% of all tweets.

We classified Twitter users into three categories: organizations, individuals, and celebrities. A user was labeled as an organization if either their screen name or profile description included one or more of the following keywords: cancer, foundation, foundtion[sic.], health, department, organization, agency, news, group society, committee, volunteer, county, government, network, firm, company, companies, nurse blog, we, promotions, marketing, forum, campaign, pharmacy, and pharmaceutical. These keywords were identified as representing organizations based on a review of Twitter profiles and screen names of users who had tweeted about breast cancer. Celebrities were users who did not match the organization criteria, but who had at least 100,000 followers and/or who had a verified Twitter account. Verified accounts are only available to high profile individuals or organizations, and ensure other users that they are following an authentic, legitimate Twitter account, not an impersonation [26]. We saw celebrities as distinct from a typical individual user on Twitter because they occupy a separate place in society’s infrastructure and they generally have more followers. All other Twitter users were categorized as individuals.

In Twitter, users may post original tweets, or they may retweet posts from others, in which case the corresponding tweet conventionally begins with “RT @*username*” or contains “via @*username*”. Retweets are a specific type of mention, in which a user re-posts content from another user. In addition to retweeting others’ posts, users may also mention others in their tweets by including their usernames using the “@*username*” syntax where *username* is the Twitter user profile name of the user being mentioned. When a user is mentioned in a tweet, that tweet is placed into a separate list in the Twitter interface (i.e. a Twitter “feed”) and generates other notifications (e.g., email message) which increases the chances of garnering the mentioned user’s attention. These mentions can serve to endorse others to some degree. Often when mentioning another user, the username will occur in the middle of a tweet, whereas, by starting a tweet with the username it is clearly directed to that user, perhaps to evoke a response. A single tweet may mention as many users as the tweeter has room to write. We classified each tweet as an original or a retweet, and then whether it included a mention.

Statistical methods included ANOVA to test for differences between celebrities, organizations and individuals and interval level variables. Chi square tests were used to test for differences with dichotomous variables. For content analysis, we turned to techniques from computational linguistics. Because they can be applied efficiently, these techniques allow researchers to consider large volumes of data, avoid expensive human coding, and discover topics automatically. We focused here on topic discovery through Latent Dirichlet Allocation (LDA) [27]. LDA is a probabilistic model that hypothesizes that each document (e.g., individual tweets) in a given corpus (e.g., all the tweets) has been generated as a mixture of unobserved, or latent, topics, where a topic is characterized by a categorical distribution over words. Hence, given a set of documents, LDA automatically produces a series of topics, or word collections, present in the documents. We used the popular MALLET implementation, an open-source software that contains the LDA algorithm [27,28]. We also prepared a list of topics associated with BCAM, as identified from an initial review of the tweets and the authors’ experience. The selected keywords are shown in Table 1. LDA produced no new keywords, providing validity to the list of topics originally generated.

**Table 1:** Key words used for content analysis of breast cancer tweets

**Category**	**Terms**
Wear pink	Wear, pink, socks, shirts, bracelet
Loved ones	Beat, survivor, grandma, mom, mamma, aunt, memory, die, story
Resentment	Other types, tired of, annoyed, resent, attention, fair
Walks & Runs	Walk, race, run, komen
Early detection	Mammogram(s), screening(s), exam, mammography, doctor, visit, detection, lump
Diagnosis	Symptom(s), diagnose(d),diagnosis
Treatments	Mastectomy, lumpectomy, chemo, radiation, chemotherapy, surgery, surgeon
Fundraising	Money, fundraiser, fundraising, donate, research, fund(s), donation, proceeds, benefit

## Results

**Figure 1:**
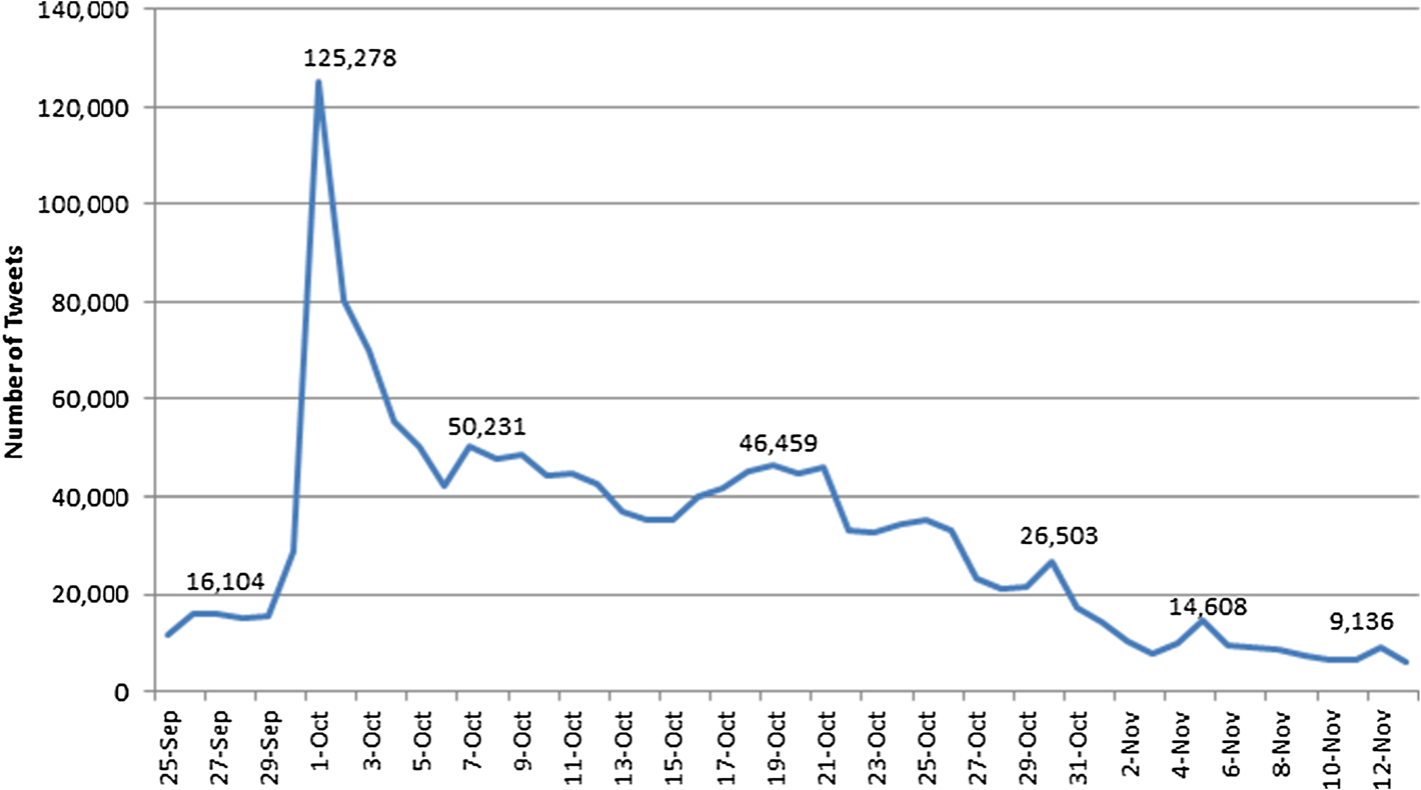
Number of breast cancer tweets during breast cancer awareness month.

There were 1,351,823 breast-cancer related tweets during BCAM by 797,827 unique Twitter users. Compared to pre-BCAM levels, the tweets spiked dramatically the first few days, with a peak of 125,278 on October 1st (Figure 1). The tweet frequency then tapered off rather quickly through the rest of the month. There was an average of 1.69 BCAM-related tweets per user made during the month.

Users tweeting about breast cancer represented individuals (93.2%), organizations (6.5%), and celebrities (0.3%). Organizations were responsible for 10.7% of all the tweets, posting an average of 2.78 times during the month. Celebrities posted an average of 2.35 times, accounting for less than one percent (0.4%) of tweets. In contrast, individuals made an average of 1.62 tweets during the month, accounting for the majority of tweets at 88.9%. The difference between the groups in the number of times the group posted breast cancer tweets was significant (f = 1193.41, p < 0.0001).

The reach of a message was estimated by calculating impressions [29], meaning the number of Twitter followers who potentially see a user’s tweet. When a user tweets, these tweets are added to the streams of those users that follow them so that the tweet may assumedly be seen and read. Impressions were calculated per user by multiplying the number of their followers by the number of BCAM tweets they made. As shown in Table 2, largely because of their greater number of followers, on average, celebrities made far more impressions than organizations, which in turn made far more than individuals (f = 5394.76, p < 0.0001). However, because the total number of individuals is much greater than that of organizations, as a group, individuals ultimately account for more impressions than organizations, and yet not as many as celebrities.

**Table 2:** Characteristics of Twitter users tweeting about breast cancer during breast cancer awareness month

		**Organizations**	**Individuals**	**Celebrities**	**All users**
		**(N = 52,109)**	**(N = 743,673)**	**(N = 2,045)**	**(N = 797,827)**
Number of Twitter followers		217,128,894	433,701,544	465,013,040	1,115,843,478
	Mean	4,166	583	227,390	1,398
	Median	286	190	50,531	194
Number of other Twitter users they are following		46,054,231	315,152,760	12,729,206	373,936,197
	Mean	883	423	6,224	468
	Median	271	216	418	218
Number of BCAM tweets		144,886	1,202,132	4,805	1,351,823
	Mean	2.78	1.62	2.35	1.69
	Median	1	1	1	1
Number of lifetime tweets*		356,490,826	5,448,8247,18	25,591,726	5,830,907,270
	Mean	6,841	7,326	12,514	7,308
	Median	1,692	2,584	5,822	2,528
BCAM impressions		764,914,959	1,005,051,671	1,258,484,973	3,028,451,603
	Mean	14,679	1,351	615,396	3,795
	Median	404	225	73,503	232

* Total number of tweets (about any topic) users have posted to date when data were collected.

The vast majority of tweets in our study were originals: 94.2% for organizations, 94.9% for individuals, and 91.0% for celebrities. As shown in Table 3, just over a quarter of tweets included a mention. Celebrity tweets were more likely to include mentions than tweets by organizations or individuals (χ^2^ = 1259.86, p < 0.0001). However, individuals were more likely to direct a tweet to a specific person, meaning the tweet began with the @ symbol. This was more than organizations or celebrities (χ^2^ = 2712.53, p < 0.0001). Thus, while celebrities are more likely to mention or endorse others, it is individuals who appear more likely to be actually engaging in discussions with others by directing their tweet to a specific Twitter user.

**Table 3:** Retweets and mentions included in breast cancer tweets

	**Organizations**	**%**	**Individuals**	**%**	**Celebrities**	**%**	**All users**	**%**
	**(N = 144,886)**		**(N = 1,202,132)**		**(N = 4,805)**		**(N = 1,351,823)**	
Original	136,439	94.2	1,140,866	94.9	4,371	91.0	1,281,676	94.8
Retweet	8,447	5.8	61,266	5.1	434	9.0	70,147	5.2
Any mention (containing @)	37,253	25.7	344,354	28.7	2,196	45.7	383,803	28.4
Directed to a user (starting with @)	10,940	7.6	145,369	12.1	324	6.7	156,633	11.6
Retweeted by others in study	11,561	7.98	34,298	2.9	4,955	103.1	50,814	3.8

**Figure 2:**
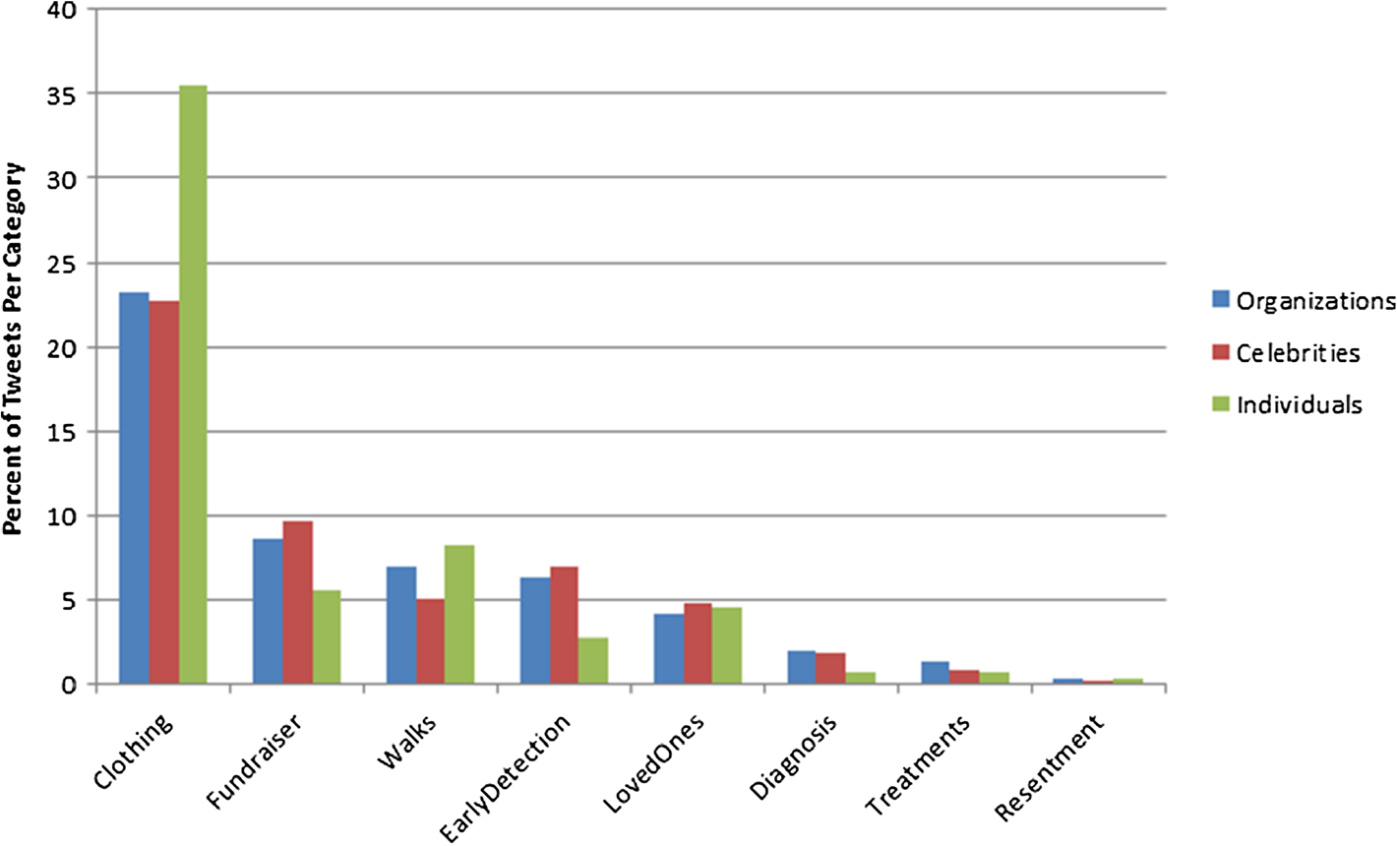
Type of breast cancer tweet by user.

Tweets most commonly contained content related to wearing pink, and promoting walks/runs and fundraisers (Table 4; Figure 2). There were differences in the content of tweets produced by organizations, celebrities, and individuals. Organizations and celebrities were similar in their content, each putting more emphasis on fundraisers, early detection, and diagnoses, than individuals did. In contrast tweets from individuals were more likely to mention clothing and walks compared to organizations and celebrities (χ^2^ = 347.38, p < 0.0001).

**Table 4:** Content analysis of tweets and retweets by type of user

	**Organizations**		**Individuals**		**Celebrities**		**All users**	
	**Tweets**	**Retweet**	**Tweets**	**Retweet**	**Tweets**	**Retweet**	**Tweets**	**Retweet**
	**N (%)**	**N (%)**	**N (%)**	**N (%)**	**N (%)**	**N (%)**	**N (%)**	**N (%)**
Total N	144,866	8,447	1,202,132	61,266	4,805	483	1,351,823	70,147
Clothing	33,591(23.3)	1,853 (21.9)	427,359 (35.6)	15,706 (25.6)	1,088 (22.6)	89 (20.5)	462,038 (34.2)	17,648 (25.2)
Fundraiser	12,567 (8.7)	560 (6.6)	66811 (5.6)	3,513 (5.7)	464 (9.7)	32 (7.4)	79,842 (5.9)	4,105 (5.9)
Walks	10,020 (6.9)	422 (5.0)	98,682 (8.2)	3,394 (5.5)	245 (5.1)	23 (5.3)	108,947 (8.1)	3,839 (5.5)
Early detection	9,096 (6.3)	623 ( 7.4)	32,532 (2.7)	2,702 (4.4)	332 (6.9)	21 (4.8)	41,960 (3.1)	3,346 (4.8)
Loved ones	6,040 (4.2)	372 (4.4)	54,769 (4.6)	2,446 (4.0)	233 (4.9)	21 (4.8)	61,042 (4.5)	2,839 (4.0)
Diagnosis	2,918 (2.0)	167 (2.0)	8,459 (0.7)	586 (1.0)	90 (1.9)	4 (0.9)	11,467 (0.9)	757(1.1)
Treatments	2,012 (1.4)	98 (1.2)	7,846 (0.70	369 (0.6)	38 (0.8)	0	9,896 (0.7)	467(0.7)
Resentment	394 (0.3)	18 (0.2)	3,443 (0.30	142 (0.2)	7 (0.2)	1 (0.2)	3,844 (0.3)	161 (0.2)

In terms of having their tweets retweeted by other Twitter users who had also tweeted about breast cancer and were our study population, celebrities were retweeted 4,955 times which is nearly the same as the total number of tweets authored by the group (103%). In contrast, organizations were retweeted 11,561 times (7.98% of the amount of total tweets) and individuals were retweeted 34,298 times (2.85% of the amount of total tweets), making tweets from celebrities much more likely to be retweeted on average (12.92 times more likely than organizations and 36.14 times more likely than individuals). The largest percentage of retweets were about clothing, as were the original tweets, though at a lower level. However, the proportion of retweets about early detection was higher for both organizations and individuals (Table 4).

In addition to using the defined categories, unsupervised content clustering was also performed on the content using LDA. Interestingly, the topics discovered by LDA reflected mostly the same frequent categories used in Table 1 (e.g., clothing, walks, fundraisers, etc.).

## Discussion

This study analyzed how individuals, celebrities, and organizations are using Twitter during BCAM to spread the breast cancer message. Results showed that tweeting about breast cancer was a singular event and not consistent throughout the month. There was a pronounced spike in breast cancer related tweets on the first day of BCAM, followed by an immediate decline, which gradually continued to fall over the course of the month. There was also a marked increase in tweets during weekends (i.e., Oct 6–7, 20–21, 27–28), correlating with awareness-related public events such as Race for the Cure.

The majority of tweets did not promote any specific behavior for prevention, including screening mammograms. There is evidence that BCAM events during the mid-1990s were effective at increasing diagnosis during November, the month following BCAM [12]. However, the impact and effectiveness of these events on screening behaviors and subsequent diagnosis has declined, possibly due to increased yearly routine screenings, rather than screening due to a BCAM event [12]. It has been suggested that rather than using national campaigns to increase screenings, that BCAM efforts be used to raise funds for breast cancer research and facilitate social support for breast cancer patients and survivors [12]. The results of our study provide evidence that during BCAM a key message being conveyed, at least through the social media platform Twitter, is that of supporting fundraising efforts.

Each of the three groups tweeted about different aspects of breast cancer. Organizations and celebrities tended to focus on promoting behavior of early detection and fundraising, while individuals focused on events and wearing pink clothing. Because most events, such as Race for the Cure, are also associated with raising money to support breast cancer research, our results suggest that all groups are using social media to spread the message about the need for more research funding. Using social media for fundraising purposes has been documented, including well-coordinated grassroots initiatives such as Twestival [30]. Additionally, Nah and Saxton found that organizations that rely on donor-based funding as compared to government funding, tended to use social media more often [31]. Therefore, use of social media by breast cancer organizations to raise funds appears appropriate.

Though the inherent value in social media is its social nature and the opportunity for dialog and two-way communication, it appears that Twitter is being used during BCAM as primarily one-way communication. The average number of tweets was less than two, meaning there are not on-going conversations between users, particularly organizations, and their followers. In addition, the majority of the messages are not retweeted, with the exception of celebrities. The lack of retweets may be because the content of the tweets are such that they are not considered of high value to retweet and share. Research suggests that the Twitter posts that get retweeted are those related to issues that are time sensitive, breaking-news, and trendy topics [32]. Based on the content analysis of the tweets during BCAM, most of the tweets were time sensitive, but not necessarily breaking-news or trendy, and therefore may not have been perceived as retweet worthy.

Another way to increase dialog on Twitter is using the @*username* in part of the tweet as a mention, as described earlier. If a tweet includes a mention, then it is more likely to be seen by a specific user instead of getting lost in the Twitter feed. Individuals were more likely to include the @username at the start of the tweet, indicating they were directing their post to a specific person. Although literature suggests that using the @ symbol and mentions increases the potential communication dialog there is limited empirical evidence about the overall effectiveness of increasing the overall amount of dialog [33]. However it seems plausible that this type of approach could increase the likelihood of a response and foster dialog about breast cancer. Organizations and celebrities were probably less likely to use mentions because they may be using Twitter as a medium to broadcast and share a one-way message with many followers.

Spokespersons or messengers are key to any successful communication campaign [34]. While word of mouth marketing has been an essential part of traditional marketing communication strategies, of electronic word of mouth (eWOM) is becoming increasingly valuable as well [35]. In addition, the influence of opinion leaders has been vital to the diffusion of innovations, with innovations encompassing ideas, practices or objects [36], which could include the idea of contributing to breast cancer research or receiving a mammogram. Messengers and opinion leaders can be celebrities or ordinary individuals [37] both of which were included in our study.

Celebrities reached more people during BCAM than did organizations or individuals, despite making fewer tweets overall. Individuals had a greater overall reach than organizations. This was due to the fact that there were a greater number of individuals than organizations tweeting about BCAM. Because of small groups of very active organizations and celebrities, the mean number of tweets was higher for these groups than for individuals.

However, the influence of celebrities and individuals may not be equal. Social network analysis states that centrality of opinion leaders is important in determining their level of influence [38]. That is, people who are more closely connected and communicate more often have greater influence [38]. In assessing the degree of influence among Twitter users, as measured by indegree (number of followers), retweets and mentions, researchers found that persons with many followers, such as celebrities, were not as influential as others who are mentioned or retweeted [39]. In contrast, our results show that celebrities were retweeted more often than individual or organizations and were more likely to include mentions. However, in our study individuals were more likely to direct a tweet to a specific user, as indicated by the @ symbol, signifying that this may be more influential in initiating conversations about breast cancer.

Organizations may want to consider framing the BCAM message to focus more directly on raising funds for breast cancer research and encouraging social support as suggested [12], while not ignoring the importance of promoting screenings. In doing so, organizations may want to consider partnering with both individuals and celebrities as spokespersons or messengers for their campaigns. To increase overall reach of messages for breast cancer and other health observances, organizations and agencies may consider partnering with high profile spokespersons that have a large number of followers. For example, celebrity, Angelina Jolie recently underwent elective surgery for a double mastectomy to reduce her risk of breast cancer [40]. Twitter messages during BCAM by a celebrity such as Ms. Jolie would have a tremendous reach.

In addition, organizations can partner with individuals to spread the message within their networks and engage followers in conversations about breast cancer. As noted earlier, Twitter use is more common among the younger population (ages 18–30), particularly among Black and Hispanic origin, though males and females tend to use it equally, as well as among those with more formal education, and higher incomes [1]. Although this age demographic is not the population most likely to develop breast cancer, their mothers and grandmothers are and are the persons who should receive screening mammograms. Furthermore, these young adults are members of the millennial generation, meaning they were born between 1980–2001. Millennials have unique characteristics that lend this group to be uniquely targeted for health awareness campaigns. Millennials are interested in maintaining connections with their families are concerned with social and environmental issues, they support causes, and they want to make a difference in the world [41]. Millennials also tend to volunteer more often and they report that their family influences their volunteerism [42]. In a study of how organizations engage with millennials, researchers found that they are more likely to donate to a cause if their friend recommended it or if they know someone affected by it [43]. Therefore, organizations may benefit from engaging these younger Twitter users in promoting fundraisings, enabling social support and awareness of breast cancer screening.

We have presented BCAM as a case study of how Twitter is used. There are several other NHO that could incorporate social media, including Twitter, into its communications strategy. Topics that are of particular relevance, due to the fact that Twitter use among young adult populations is high and the prevalence of these behaviors is also high, or young adult populations are directly affected by these issues, include: teen dating violence, sleep awareness, alcohol awareness, distracted driving, melanoma/skin cancer detection and prevention, and so forth.

### Limitations

These results should be interpreted according to the following limitations. The reach achieved during BCAM may be less due to the fact that the list of followers may not be mutually exclusive. That is, some Twitter users may be following multiple people who were all tweeting about breast cancer during BCAM. It is, of course, impossible to guarantee that all tweets are actually seen by all followers. Indeed, since new tweets are constantly added to a user’s stream, particular tweets may be drowned in the sea of data, either due to the sheer velocity of the incoming data (e.g., a user may be following many people who tweet with high frequency thus creating a very high throughput tweet stream that may be difficult to keep up with) or the frequency of inspection (e.g., a user may look at their tweet stream only occasionally so that the automatic re-actualization of the stream upon viewing causes older tweets to be pushed way back in the stream). And yet, each tweet represents a potential impression made on the follower, analogous to marketers seeking to make impressions on consumers to influence them to buy a product or service.

Clearly, the likelihood of someone seeing a message increases with the number of impressions generated. For example, if two users have the same number of followers, but the first produces twice as many tweets, then that first user is more likely to have its message seen. Similarly, if the two users produce the same number of tweets, but the first has twice as many followers as the second, then the message of the first user is also more likely to be seen.

We may have underestimated the number of tweets during BCAM. It is possible that there are other BCAM-related tweets we missed because they were not covered by our keywords. It is also possible that not all tweets were delivered to us by the Twitter interface, although that is not possible to verify.

Finally, the use of keywords to label organizations based on their profile descriptions may have missed some organizations if organizations did not provide a description of who they were. Similarly, we may have missed classifying some celebrities based on criteria for number of followers and verified accounts.

## Conclusions

Twitter is being used as a one-way communication tool to spread the message about breast cancer, particularly general awareness and fundraising. To expand the reach of the breast cancer message and maximize the potential for word-of-mouth marketing using Twitter, organizations need a strategic communications plan that includes ways to ensure on-going conversations on social media during BCAM. Organizations may want to consider collaborating with both individuals and celebrities to spread the message. Social media strategies that emphasize fundraising for breast cancer research and prevention seem particularly appropriate.

## Abbreviations

BCAM: Breast cancer awareness month; LDA: Latent dirichlet allocation; NHO: National Health Observance.

## Competing interests

The authors’ declare that they have no competing interests.

## Authors’ contributions

RT: conceived of the study, participated in the design of the study, and helped to draft the manuscript. SHB: conceived of the study, participated in the design of the study, performed the statistical analysis, and helped to draft the manuscript. GCG: conceived of the study, participated in the design of the study and helped to draft the manuscript. SR: performed the statistical analysis and helped to draft the manuscript. CRD: helped to draft the manuscript. All authors read and approved the final manuscript.

## Pre-publication history

The pre-publication history for this paper can be accessed here:

http://www.biomedcentral.com/1471-2407/13/508/prepub
